# Dorsally Comminuted Fractures of the Distal End of the Radius: Osteosynthesis with Volar Fixed Angle Locking Plates

**DOI:** 10.1155/2013/131757

**Published:** 2013-05-08

**Authors:** Paritosh Gogna, Harpal Singh Selhi, Rohit Singla, Ashish Devgan, Narender Kumar Magu, Pankaj Mahindra, Mohammad Yamin

**Affiliations:** ^1^Department of Orthopaedics and Rehabilitation, Pt. B.D. Sharma Post graduate Institute of Medical Sciences, 2/11-J Medical Enclave, PGIMS, Haryana, Rohtak 124001, India; ^2^Department of Orthopaedics, Dayanand Medical College and Hospital, Punjab, Ludhiana 141001, India

## Abstract

*Background*. Dorsally comminuted distal radius fractures are unstable fractures and represent a treatment challenge. The objective of this study was to evaluate the functional and radiological outcome of dorsally comminuted fractures of the distal radius fixed with a volar locking plate. *Patients and Methods*. Thirty-three consecutive patients with dorsally comminuted fractures of the distal end of the radius were treated by open reduction and internal fixation with AO 2.4 mm (*n* = 19)/3.5 mm (*n* = 14) volar locking distal radius plate (Synthes, Switzerland, marketed by Synthes India Pvt. Ltd.). There were 7 type A3, 8 type C2, and 18 type C3 fractures. The patients were followed up at 6 weeks, 3 months, 6 months, and 1 year postoperatively. Subjective assessment was done as per Disabilities Arm, Shoulder, and Hand (DASH) questionnaire. Functional evaluation was done by measuring grip strength and range of motion around the wrist; the radiological determinants were radial angle, radial length, volar angle, and ulnar variance. The final assessment was done as per Demerit point system of Saito. *Results*. There were 23 males and 10 females with an average age of 44.12 ± 18.63 years (18–61 years). Clinicoradiological consolidation of the fracture was observed in all cases at a mean of 9.6 weeks (range 7–12 weeks). The average final extension was 58.15° ± 7.83°, flexion was 54.62° ± 11.23°, supination was 84.23° ± 6.02°, and pronation was 80.92° ± 5.54°. Demerit point system of Saito yielded excellent results in 79% (*n* = 26), good in 18% (*n* = 6), and fair in 3% (*n* = 1) patients. Three patients had loss of reduction but none of the patients had tendon irritation or ruptures, implant failure, or nonunion at the end of an one-year followup. *Conclusion*. Volar locking plate fixation for dorsally comminuted distal radius fractures results in good to excellent functional outcomes despite a high incidence of loss of reduction and fracture collapse.

## 1. Introduction

With an incidence of about 17% of all fractures, the distal radial fracture is the most common fracture in the human skeleton [1]. Dorsally comminuted fractures are highly unstable, and closed reduction results in unsatisfactory outcome [2]. There has been a consensus on open reduction and internal fixation of these fractures so as to restore the distal radius anatomy and regain early return to activity [3]. Dorsal approach allows direct articular visualization and placement of plate as a dorsal buttress; however, a high incidence of hardware-related problems has culminated in a recent trend towards volar plates [3, 4]. Volar locking plate provides stable fixed angle support that permits early active wrist rehabilitation, direct fracture reduction, and fewer soft tissue and tendon problems [3]. Recently performed studies have found it to be effective in treating dorsally comminuted distal radius fractures [4, 5]. The present study was done to evaluate the outcome of the unstable dorsally comminuted fractures of the distal end of the radius managed with a locked volar plate.

## 2. Patients and Method

This prospective study is comprised of 33 consecutive patients with dorsally comminuted fractures of the distal radius. Exclusion criteria were skeletally immature patients, open fractures, pathological fracture, and previously operated or nonfunctional wrist. Fractures were classified as per AO/OTA classification (Table 1). Dorsally displaced fracture fragments were indirectly reduced using a combination of direct pressure and ligamentotaxis (the intact dorsal capsule aids in falling back of the boney fragments). Fractures were fixed using volar approach of Henry with 2.4 mm (*n* = 19)/3.5 mm (*n* = 14) volar locking distal radius plate (Synthes, Switzerland, marketed by Synthes India Pvt.) under image intensifier. The stability of fixation was checked by moving the wrist joint through the complete range of motion. Postoperatively Plaster of Paris backslab was given to give rest to the operated part. Active finger movements were encouraged immediately after operation along with elevation.

Supervised physiotherapy in the form of active wrist movements and hand functions pinch and grasp was begun at 2 weeks after suture removal. Patients were reviewed clinical-radiologically at 6 weeks 3 months, 6 months and 1 year postoperatively. Subjective assessment was done as per DASH questionnaire. Functional evaluation was done by measuring range of motion around the wrist, whereby extension, flexion, supination, and pronation were measured with the help of goniometer. Grip strength was measured in Kg with the help of dynamometer and compared with the contralateral side. Paired Student's *t*-test was applied to compare the functional parameters, namely, extension, flexion, supination, pronation, and grip strength at 6 weeks and at 1 year. The radiological determinants, that is, radial angle, radial height, volar angle, and ulnar variance were measured with the help of goniometer on standard anterioposterior and lateral radiographs of the wrist. Satisfactory reduction was defined as <10° of dorsal tilt, <2 mm of radial shortening and <1 mm of articular incongruity [4, 6, 7]. Paired Student's *t*-test was applied to compare the radiological parameters, namely, radial angle, radial length, volar angle, and ulnar variance measured in the immediate postoperative period with those at 1 year. The final outcome was evaluated on the basis of functional and radiological demerit point system of Saito [8] and complications if any were noted.

## 3. Results

 There were 21 males and 5 females with an average age of 44.12 ± 18.63 years (18–81 years). The majority of cases were due to road traffic accident in younger age group, whereas direct fall on outstretched hand was a common mode of injury in the older age group. Clinicoradiological consolidation of the fracture was observed in all cases at an average of 9.6 weeks (7–12 weeks).

**Figure 1:**
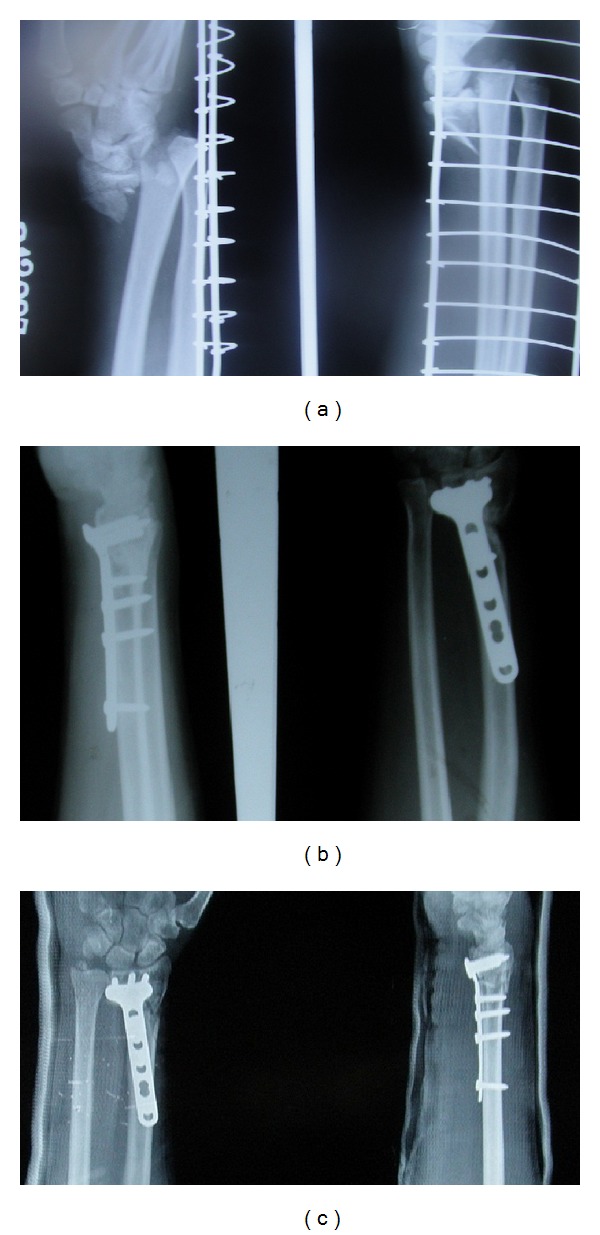
(a) Preoperative radiographs of an AO type A3 fracture of the distal end of the radius in a 53-year-old male. (b) Postoperative radiographs showing adequate reduction. (c) Radiographs at the final followup, showing that the fracture has united and the radiological parameters are maintained.

**Figure 2:**
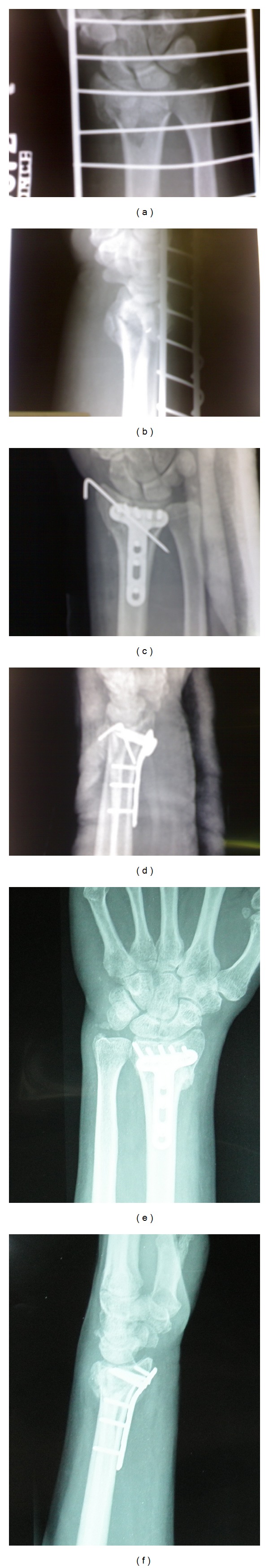
(a) Anterioposterior and (b) lateral views of wrist of a 35-year-old male with an AO/OTA type C3 fracture of the distal radius. (c), (d) Adequate reduction was achieved and open reduction and internal fixation of the fracture was done with volar locking plate and a K wire. (e), (f) Radiographs at the final followup showing union of the fracture. The locking plate was able to hold reduction till consolidation.

We were able to attain adequate reduction in all patients (Figures 1 and 2). Immediate postoperative radiological parameters were compared with those at 1 year using Student's paired *t*-test, which revealed the difference to be insignificant (*P* > 0.05) for all four parameters (Table 2). On comparing results from 6 weeks to those at 1 year, there was significant improvement in all the five functional parameters (Table 3). At 1-year followup, the mean DASH score was 16 and Demerit point system of Saito yielded 79% (*n* = 26) excellent, 18% (*n* = 6) good, and 3% (*n* = 1) fair result. Three patients had loss of reduction at subsequent followup; however, none of our patients had tendon irritation/rupture, implant failure, or nonunion.

## 4. Discussion

The importance of restoring the anatomical alignment and articular congruity is well recognised in the management of distal radial fractures [3]. Lafontaine in his study of 112 consecutive patients with fracture of the distal end of the radius suggested that the distal radius fractures with dorsal comminution are highly unstable and tend to suffer redisplacement following closed reduction [2]. Mackenney et al. reported that early instability was six times more common in fractures with any form of dorsal comminution [9]. 

Open reduction and internal fixation which allow better restoration and preservation of distal radius radiological parameters are recommended for this subset of unstable distal radius fractures [3, 10]. Significant controversy exists about whether dorsal or volar plating is superior for fixation of dorsally comminuted distal radial fractures [11–13]. 

Though dorsal approach allows for direct articular visualization and placement of internal fixation as a dorsal buttress, allowing earlier return to activity, the outcomes tend to level off with time [14]. Prominent dorsal implant, extensor tenosynovitis, and rupture have been reported as complications after dorsal plating plate. Moreover, dorsally placed implants have increased thickness of the plate, raised screw heads, and they lack the ability to contour the plate to fit the bone [15, 16]. Though the advent of low-profile dorsal plates has solved this concern to some extent [11, 16], the very approach often requires dissection of the extensor retinaculum, and sometimes resection of the Lister's tubercle.

In the volar approach, the pronator quadratus acts as a barrier by covering the distal edges of the plate, thereby minimising irritation to flexor tendons. As the volar cortex is relatively broad and flat as compared to the dorsal cortex of the distal radius, application of the volar locked plate is easy.

In a biomechanical study on dorsally comminuted extra-articular distal radial fractures comparing dorsal T-plate with volar locking plate, it was inferred that there was no difference in any of the biomechanical parameters, namely, stiffness, fragment displacement at 500 cycle-intervals, and axial load to failure between these constructs [13].

 Volar plating preserves vascular supply to dorsal metaphyseal fragments and provides steady configuration, requiring less extensive dissection and thereby resulting in a low incidence of complications. In addition, the use of the volar approach is advantageous for restoring accurate rotational alignment because the volar surface of the distal radius is usually not comminuted. Anatomical reduction of the palmar cortex may also avoid the shortening of the radius, which is important to restore the radial length [17, 18]. The locking plate system provides a more secure and reliable fixation for comminuted fractures. The fixed plate screw construct with multiple screw options for easy application in distal complex fractures provides angular stability [3]. In a biomechanical study, it was found that volar locking provides significant resistance to fracture gap motion as compared to nonlocking plates [12].

 We found that volar locking plates were able to attain and maintain good radiological and functional results (Tables 2 and 3), which supports the results in other series [4, 5, 19]. At final followup the average DASH score in the present series was 16 which is in consensus with other series [4]. Murakami et al. reported 83% excellent and 17% good outcome in their series of 24 patients of unstable distal radius fractures fixed with volar locking plate [5]. Rozental and Blazar reported 66% excellent and 44% good results in their series of 41 cases of dorsally displaced unstable fractures of the distal radius [4]. The results of our study are in consensus with them. 

**Figure 3:**
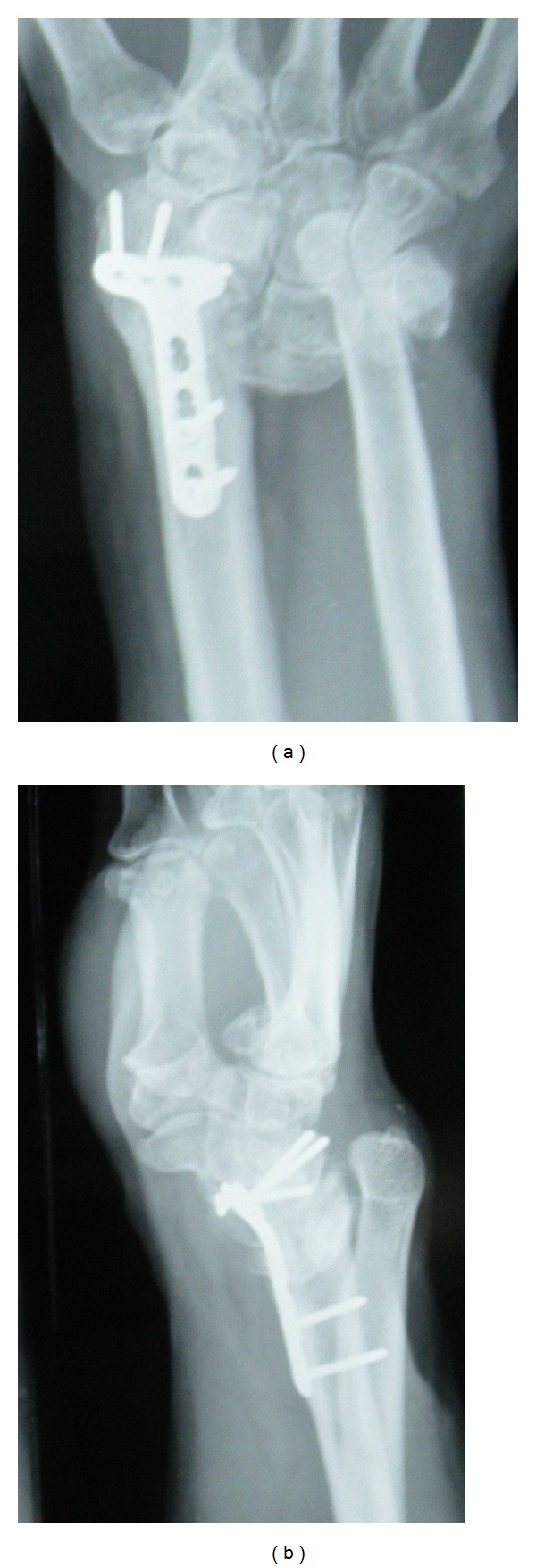
(a), (b) Radiographs at follow-up visit of an AO type C3 fracture of the right wrist which was fixed with volar locking plate. Though we were able to attain adequate reduction for the distal radius, the associated DRUJ (distal radioulnar joint) injury was missed by us. These radiographs show loss of reduction with dorsal subluxation of the carpus and DRUJ disruption.

 In our series we had 3 cases of loss of reduction subsequently leading to malunion. The first case was a 33-year-old male who sustained AO type C3 fracture of the right wrist, with associated DRUJ (distal radioulnar joint) injury. This was the first case of the series and hence it formed a part of our learning curve. We were able to obtain an adequate reduction for the distal radius; however, the DRUJ injury was missed by us. The radiographs obtained at subsequent follow-up visits revealed loss of reduction with dorsal subluxation of the carpus and DRUJ disruption (Figure 3). The fracture united but with deformity. At the final followup, the flexion extension arc was significantly less 52° though the rotations and grip strength were not affected much. This patient had a DASH score of 22. We, therefore, stress to the need to always check for the DRUJ injury in every case of distal radius fracture, as a missed DRUJ injury can be a disaster for both the patient and the doctor.

The second case too was of an AO type C3 fracture in an eighteen-year-old male patient. Due to high-grade comminution, only one screw could be placed in the distal horizontal part of the plate. The initial radiographs confirmed adequate reduction. Subsequent X rays revealed that there was dorsal collapse of the fracture, which resulted in fracture healing in a residual dorsal tilt. The third case was again was an AO type C3 fracture in a 47-year-old male patient; there was dorsal settling of the fracture resulting in fracture healing in residual dorsal tilt. Despite this, the functional outcome remained favorable in these two patients, and the patients accepted the deformity well.

The 9.09% incidence of loss of reduction in our series is comparable to that of Rozental and Blazer who reported, 4 instances of loss of reduction in 41 patients (9.75%) and Orby et al. loss of reduction in 3 out of 24 patients (12.5%) treated with volar locking plates [4, 19]. In the present series we did not have any case of hardware related complications or non-union.

When compared with previous reports on dorsal plating, volar plates appear to have a higher incidence of fracture collapse but a lower rate of hardware-related complications. Rozental and Blazer compared thier results of locked volar plating with those of dorsal plating for comminuted distal radius fractures. They identified that the overall complication rate in the volar plate group was 22% as compared to 32% in the dorsal plate group. Further, the volar plate cohort had a 10% rate of loss of reduction while the dorsally plated patients did not experience any loss of reduction, malunion, or nonunion. They found a statistically significant difference in the rate of soft-tissue complications between the 2 groups; the volar plate group had a 7% rate of hardware removal secondary to tendon irritation while the dorsal plate group had a 32% rate of hardware-related tendon complications [4, 20].

We had our own set of limitations which included a small subset of patients and a relatively short followup of 1 year. Though studies have indicated that fragment displacement that occurs does so in the early periods of motion [13], still we intend to incorporate more patients and follow up these patients for a longer period. Another drawback is that it is a longitudinal study, not a comparative one; however, we have made an attempt to highlight the aspects of both dorsal and volar plating and compared the results with the literature. To conclude, patients with unstable, dorsally comminuted fractures of the distal radius treated with volar fixed angle plate have good to excellent functional outcomes despite a high incidence of loss of reduction and fracture collapse. This technique has its own learning curve, and it is highly recommended to check for associated DRUJ injuries. Our early results with this technique are fruitful, and we recommend it for the fixation of dorsally comminuted distal radius fractures.

## Figures and Tables

**Table 1:** Clinical profile of patients.

Mean age at presentation	44.12 ± 18.63 years (range 18–61 years)	

Sex	Male	23
	Female	10

Mode of injury	RTA	26
	Fall	7

Type of fracture	A3	7
	C2	8
	C3	18

RTA: road traffic accident.

**Table 2:** Radiological evaluation.

	Postoperatively	At 1 year
Radial inclination	23.42°± 3.65°	23.98° ± 4.21°
Radial length	12.51 ± 2.77 mm	12.63 ± 2.34 mm
Volar angle	5.86° ± 6.74°	5.54° ± 7.52°
Ulnar variance	−0.77 ± 0.88 mm	−0.55 ± 1.1 mm

mm: millimeters.

**Table 3:** Functional evaluation.

	6 weeks	1 year
Extension	44.16° ± 7.21°	58.15° ± 7.83°
Flexion	42.23° ± 9.62°	54.62° ± 11.23°
Supination	76.95° ± 4.11°	84.23° ± 6.02°
Pronation	72.10° ± 6.02°	80.92° ± 5.54°
Grip strength		
Absolute value in Kgs	10.62 ± 2.88 kg	21.53 ± 3.42 kg
As a percentage of c/l side	44.26 ± 8.7%	92.26 ± 2.1%

Kgs: kilograms; c/l: contralateral.
